# Examining cultural drifts in artworks through history and development: cultural comparisons between Japanese and western landscape paintings and drawings

**DOI:** 10.3389/fpsyg.2014.01041

**Published:** 2014-09-19

**Authors:** Kristina Nand, Takahiko Masuda, Sawa Senzaki, Keiko Ishii

**Affiliations:** ^1^Culture and Cognition Lab, Department of Psychology, University of AlbertaEdmonton, AB, Canada; ^2^Department of Human Development, University of Wisconsin-Green BayGreen Bay, WI, USA; ^3^Department of Psychology, Kobe UniversityKobe-shi, Hyogo-Ken, Japan

**Keywords:** culture, Japan, Canada, artworks, masterpieces, history, development

## Abstract

Research on cultural products suggest that there are substantial cultural variations between East Asian and European landscape masterpieces and contemporary members' landscape artwork (Masuda et al., 2008c), and that these cultural differences in drawing styles emerge around the age of 8 (Senzaki et al., 2014b). However, culture is not static. To explore the dynamics of historical and ontogenetic influence on artistic expressions, we examined (1) 17–20th century Japanese and Western landscape masterpieces, and (2) cross-sectional adolescent data in landscape artworks alongside previous findings of elementary school-aged children, and undergraduates. The results showed cultural variations in artworks and masterpieces as well as substantial “cultural drifts” (Herskovits, 1948) where at certain time periods in history and in development, people's expressions deviated from culturally default patterns but occasionally returned to its previous state. The bidirectional influence of culture and implications for furthering the discipline of cultural psychology will be discussed.

## The cyclical nature of culture and psyche

Since cultural psychology has launched under the assumption that culture and psyche mutually construct one another in that our cultural meanings and practices bring rise to culturally specific ways of thinking and behaving, which in turn maintain culture (Bruner, 1990; Markus and Kitayama, 1991; Shweder, 1991), numerous studies have demonstrated that there are systematic cultural variations in cognition and perception. Specifically, members of East Asian cultures tend to be holistic in their thinking patterns, attending to and interpreting a given event contextually and as a whole, whereas members of North American cultures tend to be analytic thinkers, selectively attending to focal objects, and events independent from context and interpreting a given event by focusing on salient information (Nisbett et al., 2001; Nisbett, 2003; Nisbett and Masuda, 2003; Nisbett and Miyamoto, 2005). This heightened awareness to context results in East Asians, in comparison to North Americans, being sensitive to not only focal objects but also surrounding contextual information (Ji et al., 2000; Masuda and Nisbett, 2001, 2006; Kitayama et al., 2003; Chua et al., 2005; Masuda et al., 2008a,b, 2012a; Senzaki et al., 2014a).

In addition to the investigation of cultural influences on basic psychological processes, notably attention, researchers have recently begun to investigate the other path in how people convey dominant cultural messages by producing cultural products—public, shared, and tangible representations (Morling and Lamoreaux, 2008). Their studies have demonstrated that East Asian cultural products such as landscape drawings (Masuda et al., 2008c; Senzaki et al., 2014b), the amount of information in conference posters and websites (Wang et al., 2012), and the physical environment of cities and towns (Miyamoto et al., 2006) contain more information that represents interdependence and a holistic way of understanding the world, whereas Western cultural products contain information that represents independence and an analytic way of understanding the world.

In particular, Masuda et al.'s (2008c) studies are regarded as the first comprehensive attempts to investigate the relationship between culture and aesthetics. Historically speaking, East Asian and European cultures utilized different artistic methods in order to portray information from a three-dimensional world onto a two-dimensional canvas. East Asians see space as more flexible and all-encompassing while Westerners think of space as contained, distinguished by the separation between the ground and the sky (Vogt, 2013). Therefore, East Asian landscape art has historically applied a bird's-eye perspective in order to illustrate an entire scene. This perspective resulted in the horizon line being located high in the frame and the viewer looking down onto a scene that could be appreciated from any point of view. Furthermore, this perspective provided abundant space to allow artists to draw not only focal events, but also contextual events. In contrast, the technique of linear perspective was invented by Europeans during the Renaissance. Linear perspective allowed the artists to create an illusory three-dimensional view, where depth of field was actualized through converging information in the frame into a single point (Kubovy, 1986). However, this technique resulted in horizon lines being placed in the lower part of the frame, and one's perspective was fixed at the viewer's eye-level. Consequently, contextual information was restricted to what was realistically seen by the illustrator.

By analyzing the ratio of the horizon drawn to the frame and the number of objects used, which are useful indicators to indirectly measure people's degree of context sensitivity, Masuda et al. (2008c) identified that 15–19th century landscape masterpieces produced by East Asians were more likely than their Western counterparts to have higher horizons in the frame, contain more pieces of information, and holistically encompass context, and that this cultural variation in artistic expressions was observable even in landscape drawings of contemporary East Asian international students and American undergraduate students. Furthermore, Masuda et al.'s (2008c) studies and subsequent research (Wang et al., 2012) has demonstrated that people indeed prefer artistic expressions which reflect dominant cultural meaning systems—East Asian's context sensitive ideologies vs. Western object-oriented ideologies. These findings suggest that one's aesthetic expression and its cultural products, such as drawings, can be a useful tool to size up dominant messages of a given culture. As Bruner (1990), Shweder (1991), and Miller (1999) emphasized, one of the most important theoretical assumptions of cultural psychology is to treat culture and the human psyche as a mutually constitutive dynamism. A series of research on culture and aesthetics provide evidence that such assumptions of mutuality are empirically testable, and that cultural variations in aesthetics are substantial (Masuda et al., 2012b, for review).

## The dynamic nature of culture

Recent advances of research on culture and psychology, however, revealed that the existing model of culture and psychology is vulnerable to cultural change and that in fact, culture is not static. Researchers who advocate the importance of cultural change have demonstrated evidence of dynamic shifts in social structures in a given culture, while highlighting the discord between new social structures and the human mind in accommodating to these changes, and the potential for consequential social problems (Hamamura, 2012; Norasakkunkit et al., 2012). Nonetheless, it is also true that some cultural phenomena persist in the face of change (Richerson and Boyd, 2005; Heine, 2011). In order to overcome the lack of methodology in cultural psychology to capture culture as a dynamic processes where both change and persistence are substantial, several theorists have attempted to incorporate wider time frameworks into their theories (Tomasello, 1999; Chiu and Hong, 2006; Masuda, 2014).

For example, Tomasello (1999), in reference to theoretical frameworks of Vygotskian traditions (Vygotsky, 1978), maintained that to understand the cultural origin of human cognition, comprehensively understanding three time frameworks is necessary: phylogenetic, historical, and ontogenetic. *Phylogenetic processes* should be understood in the widest time framework. Throughout the evolution of the human species, culture has constantly influenced human biology and psychology such as conformity to the group, self-other distinction, and theory of mind. *Historical processes* focus on how cultural learning provides humans with skills for both accumulating and building on knowledge over generations through creating major and minor improvements to our cultural artifacts and behavior. This way of sustaining cultural knowledge specific to the human species is termed “*the ratchet effect”* (Tomasello et al., 1993). Finally, *ontogenetic processes* should be understood in the narrowest time framework. Children develop in the midst of cultural products and through interaction with mature members of a given culture. Throughout their entire developmental trajectory, they acquire and internalize specific skills necessary for survival in their culture. Here, examining how children interact with their caregivers, how culture is transmitted and how it is internalized, otherwise known as “*scaffolding processes*” (Wood et al., 1976), is necessary in order to depict cultural transmission processes (Richerson and Boyd, 2005).

Analyses of phylogeny require a research paradigm to examine the biological bases of human nature in a larger time frame. Therefore, it may not be applicable for most of the issues discussed in current cultural psychology. Historical and ontogenetic processes, however, have the potential to be incorporated into current research paradigms in cultural psychology. In fact, several studies have addressed the issues under the name of culture and history as well as culture and development.

### Culture and history

Compared to research on ontogenetic processes, research on historical processes has not been fully examined in psychology. In the limited research that exists, studies on historical changes in self-esteem (Twenge and Campbell, 2001; Twenge et al., 2012) must be counted as successful examples. Much research on historical trend analyses of human behaviors has been done in the field of political sciences (e.g., Putnam, 2000), census analyses, and demographic analyses (e.g., Goldin, 1998), and research on intelligence (e.g., Flynn, 1987, 1994, 1999). Furthermore, although the field of art history has addressed artistic expressions throughout time (e.g., Giedion, 1964; Gombrich, 1966), few research has been done in cultural psychology. To answer the necessity of historical research on culture and aesthetics, as aforementioned, Masuda et al.'s (2008c) historical analyses examined landscape masterpieces spanning 500 years, and demonstrated systematic cultural variations in artistic expressions between East Asians and Westerners.

### Culture and development

Along the reasoning of Tomasello (1999) and Vygotsky's (1978) theoretical frameworks, cultural psychologists recently have investigated developmental processes which lead children to acquire culturally dominant knowledge, and answer the questions of how and when these differences emerge in their developmental trajectory. Generally, these findings have demonstrated that cognitive differences between cultures emerge in early elementary school (Duffy et al., 2009; Kuwabara et al., 2011; Kuwabara and Smith, 2012; Imada et al., 2013), and through interaction with their children, caregivers convey culturally important messages, which may be the bases of culturally specific patterns of attention (Fernald and Morikawa, 1993; Senzaki et al., 2014c). Research on culture and aesthetics in a developmental context has also demonstrated that aesthetic expressions are systematically different across cultures (Rübeling et al., 2011; Gernhardt et al., 2013; Ishii et al., 2014). In line with these findings, Senzaki et al. (2014b) examined cultural variations in landscape artworks produced by primary school children in Japan and Canada, and demonstrated that once children understood the concept of a horizon (age 8 for both cultures), Japanese children drew the horizon higher in both studies and integrated more objects in their collages than did Canadian, the pattern of which is consistent with that of young adult data (Masuda et al., 2008c).

## Objectives and hypotheses

The historical and ontogenetic research on culture and aesthetics provide us evidence that cultural variations in aesthetic expressions are substantial. However, these studies entail some critical drawbacks. First, in Masuda et al.'s (2008c) historical analyses, the data was grouped together, therefore not considering whether cultural patterns of perception remained consistent throughout all time periods and historical circumstances. In addition, they failed to include masterpieces in a very important historical period. Japan did not engage in the importation or exportation of goods with different countries and was essentially closed from the early 17th century until the Meiji Restoration in the late 19th century, 1868 (Pollack, 2008; Rimer, 2012). However, the Meiji government actively endorsed utilizing Western systems not only to overhaul political, military, technology, and education systems, but also in the arts in order to “modernize” the country. Therefore, there is a possibility that Japanese artistic expressions shift toward that of Westerners during this period. This change created a ricochet in the West as well. After the Meiji Restoration, Japanese artworks strongly influenced Western arts. Under the name of *Japonisme*, for example, European artists, especially those who were in France, incorporated the flat and two-dimensional artistic styles of the Japanese into their artworks (Ives, 1974). Impressionists were also strongly influenced by traditional Japanese artwork (Sullivan, 1989; Walker, 2008), which led them to radically create new types of expressions, denying traditional linear-perspectives. In light of historical events in the late 19th century, more comprehensive historical analyses are needed to examine whether cultural changes in psyche occur in the course of history in Japanese and Western arts. In sum, examining data from an extensive time period, such as centuries, is essential in order to account for economic, politic and sociodemographic changes and its potential impact on psychological tendencies (Rice and Steele, 2004).

Second, in Senzaki et al.'s (2014b) ontogenetic analyses, the target populations were elementary school children and undergraduate students, missing the data of adolescents. Adolescence is a transitional stage from childhood to adulthood and individual identity development becomes the central developmental task, which involves experimentation and establishing the self as independent from caregivers (Erikson, 1968; Kroger, 2007). Furthermore, in some cognitive domains adolescents have been found to be instigators of dramatic change, such as in language, through modification and having larger peer groups to transmit and reinforce the changes made (Kerswill, 1996). As a result, adolescent patterns of behavior may drift from their child and adult counterparts.

To address these issues, we conducted two studies, one from an historical perspective, and one from an ontogenetic perspective. In Study 1, we examined whether culturally unique patterns of perception in artwork remained consistent throughout history or are subject to change through cultural exchange during the late 19th century, by comparing overall trends of the location of horizon in Japanese and Western landscape artwork from the 17th century through 20th century. In Study 2, we examined perceptual patterns in cultural products throughout development in order to determine whether cultural drifts occur during adolescence. Specifically, we investigated perceptual styles in how adolescents and adults in Japan and Canada created landscapes using both drawing (Study 2a) and collage (Study 2b) mediums. Within these artworks, we focused on horizon height, the number of objects, and the area covered by the objects in order to determine context-inclusiveness.

Because of the nature of exploratory investigation, potential changes in aesthetic expression will be treated as a result of *cultural drift*. The concept of cultural drift has been used in anthropology as a form of cultural change similar to evolution (Herskovits, 1941, 1948; Eggan, 1963), resulting from institutional, political and social change. In the current research, cultural drift will refer to gradual deviations from culturally-specific psychological tendencies throughout history based on modifications and improvements in artifacts and tools made by each generation. These drifts may occur as a result of cultural exchange, finding new trends within another culture's aesthetic products and integrating the new knowledge into existing cultural frameworks. We examined historical and ontogenetic trends in artistic expressions by contrasting two competing hypotheses: the “Resilience to Change” hypothesis vs. the “Cultural Drift” hypothesis. The “Resilience to Change” hypothesis maintains that cultural changes would not be observed after 19th century (Study 1) nor during adolescent periods (Study 2). In contrast, the “Cultural Drift” hypothesis maintains that there are substantial cultural changes observed after 19th century (Study 1) and during adolescent periods (Study 2). We also discussed whether the changes, if any, stabilize or continue to drift.

### Study 1: historical landscape masterpieces

In order to investigate the process of cultural drift, Study 1 examined Japanese and Western historical landscape masterpieces from the 17 to 20th centuries using similar methodology as Masuda et al. (2008c). We especially attempted to identify changes in trends while dividing masterpieces based on the period of production and taking into account the initial influence of the Meiji Restoration in the late 19th century, and subsequently in the early and later 20th century.

#### Methods

***Materials***. Seventeenth to twentieth century Japanese landscape art pieces (*n* = 619) from Japanese and Western art museum online databases and art books, and European landscape art pieces (*n* = 761) from Western art museum online databases and art books, were compiled and examined (see Appendix A in Supplementary Material). Given that our target of analysis was to determine whether cultural drifts occurred following the Meiji Restoration in the late 19th century, and the limitability of Japanese landscape art in the 17th century, we grouped 17 and 18th century data by every 100 years (1600–1699, *n* = 242; 1700–1799, *n* = 232) and the 19 and 20th century data by every 50 years (1800–1849, *n* = 190; 1850–1899, *n* = 233; 1900–1949, *n* = 297; 1950–1999, *n* = 186)^1^.

#### Results

Similar to Masuda et al. (2008c), horizon height in the landscape art pieces was the target of analysis. Two research assistants blind to the hypothesis (Coders 1 and 2) and the primary investigator (Coder 3) measured the location of the horizon using a coding guideline developed by the first author (see Appendix B in Supplementary Material). Coder 1 coded 2/3 the European art and Coder 2 coded all of the Japanese masterpieces and 1/3 of the European masterpieces. To ensure that the developed guideline would also apply to historical landscape art created by established artists, the primary investigator (Coder 3) coded all of the art pieces for both cultures. The interrater agreement for the horizon height was 85% for the Japanese masterpieces (Coders 1 and 3). For European masterpieces, it was 97% for Coders 1 and 3, and 98% for Coders 2 and 3.

**Figure 1:**
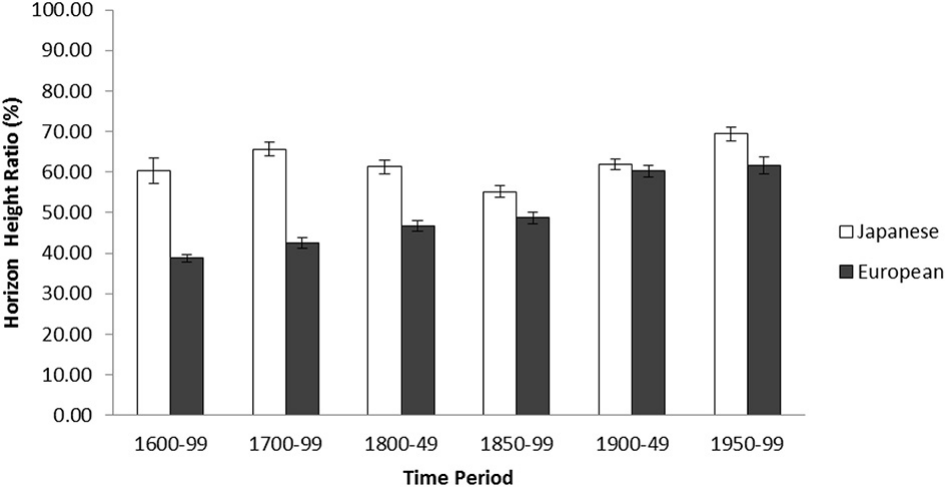
**Average horizon height ratio in percentage by time period for Japanese and European historical landscape artwork (1600–1999) in Study 1**. The error bars represent standard error.

A 2 (Culture: Japanese Arts vs. Western Arts) × 6 (Time Period: 1600–1699, 1700–1799, 1800–1849, 1850–1899, 1900–1949, and 1950–1999) ANOVA was applied to the horizon height ratio of the historical landscape art. There was a significant main effect of culture, *F*_(1, 1368)_ = 179.05, *p* < 0.001, η^2^_p_ = 0.116 as Japanese historical landscape artwork had higher horizons (*M* = 62.55, *SD* = 17.58) than that of European landscapes (*M* = 48.46, *SD* = 17.64). There was also a main effect of time period, *F*_(5, 1368)_ = 26.57, *p* < 0.001, η^2^_p_ = 0.089; however, this pattern was qualified by an interaction between culture and time period, *F*_(5, 1368)_ = 15.88, *p* < 0.001, η^2^_p_ = 0.055. The simple effect analyses revealed that there were significant cultural variations between 1600–1699, *t*_(1368)_ = 7.66, *p* < 0.001, between 1700–1799, *t*_(1368)_ = 10.89, *p* < 0.001, between 1800–1849, *t*_(1368)_ = 6.11, *p* < 0.001, and between 1850–1899, *t*_(1368)_ = 3.04, *p* < 0.01. During 1900–1949, in contrast, there were no cultural differences, *t* < 1, *ns*^2^. Cultural variations, however, emerged again between 1950–1999, *t*_(1368)_ = 3.29, *p* < 0.001. In addition, the location of horizon in Japanese artwork during 1850–1899 was marginally lower compared to the period of 1600–1699, *t*_(1368)_ = 1.74, 0.05 < *p* < 0.10, and significantly lower compared to the period of 1700–1799, *t*_(1368)_ = 4.88, *p* < 0.001, the period of 1800–1849, *t*_(1368)_ = 2.71, *p* < 0.01, the period of 1900–1949, *t*_(1368)_ = 3.36, *p* < 0.001, and the period of 1950–1999, *t*_(1368)_ = 6.35, *p* < 0.001, showing a significant drop in the location of horizon during the Meiji Restoration. In contrast, the location of horizon in Western masterpieces historically continued to show gradual increase as evident that the horizon height of the period of 1700–1799 was higher than that of the period of 1600–1699, *t*_(1368)_ = 2.00, *p* < 0.05, and that of the period of 1900–1949 was higher than that of the period of 1850–1899, *t*_(1368)_ = 5.82, *p* < 0.001 (Figure 1).

#### Discussion

Replicating Masuda et al.'s study (2008c), Study 1 demonstrated that, overall, the location of horizon in masterpieces produced by Japanese artists were higher than that of Western artists. However, supporting the “cultural drift” hypothesis, there were substantial changes in artists' expressions especially after the late 19th century. Horizons in Japanese masterpiece landscapes were significantly lower from 1850–1899 in comparison to earlier time periods but began increasing again from the 1900s. We interpreted that this pattern was observed due to the change in policy before and after Meiji Restoration in 1868. As aforementioned, during this period, the Japanese government endorsed Western systems to modernize society.

In particular, the government established the Technical Art School (*Kobu Bijutsu Gakkou*) in 1876, where “Yōga” [Western Art] courses were taught by European artists, and young and future-renowned Japanese artists learned to draw Western-style landscape images (Yamanashi, 2012). However, soon after, aesthetic nationalists such as Fernollosa (Yamanashi, 2012) and Okakura Tenshin (Clark, 2012), emphasized the rediscovery and maintenance of traditional Japanese art, which was perceived to become eventually lost through Westernization (e.g., Barber, 1995; Sam-Sang, 2011; Rimer, 2012). In contrast, the data for European horizon location did not demonstrate this kind of vacillation. One reason for this difference may be that Japanese were forced to learn and adopt Western painting styles as part of governmental policy during the Meiji Restoration, whereas Western artists adopted Japanese techniques of their own accord (Sullivan, 1989). Although the location of horizon is just one parameter of artistic expressions, the results clearly depicted the curvilinear vs. linear trends of cultural drift on top of robust cultural differences.

Similarly, horizons in Western landscapes drastically changed from the 1900s, becoming higher than that of traditional masterpieces. We attribute this cultural drift to the results of *Japonisme* and Impressionists' motivation to become free from the constraints of traditional linear-perspective, which bind the viewer's standpoint onto a single spot, and to invent an alternative expression in landscape arts. Interestingly, the change in the trend in Western arts continued throughout the subsequent time periods—the height of horizon increased in a linear pattern. We interpreted that, since the challenge of impressionism, modern and recent artists such as Salvador Dali, Georgia O'Keefe, and Wayne Thiebaud all showed their eagerness to discover new expressions beyond the status quo. However, it is unknown whether the trend will return to portraying lower horizon locations.

Finally, it is note-worthy that general patterns of cultural variations in the location of horizon were observed again in the late 20th century data. We interpret that although cultural drifts produced a variety of changes in artistic expressions, on top of the dynamic process, there is for certain room for people to rediscover their traditional ways of artistic expressions, which results in maintaining substantial cultural variations in artistic expressions. We speculate that the “resistant to change” effect is maintained beyond the artists' will. The dominant patterns of attention is internalized in the early part of the developmental trajectory, and strongly binds with other types of social cognition such as attitude inference, causal attributions, reasoning styles (e.g., Nisbett et al., 2001) and basic perception, notably attention (Nisbett and Masuda, 2003).

In sum, historical analyses in Study 1 demonstrated both resilience to change and cultural drifts. Culture is constantly changing with the East and West influencing each other bidirectionally. At the same time, once an artistic expression becomes dominant in a given cultural milieu such an initial state can still be a powerful source to maintain culturally specific trends in artistic expression.

### Study 2a: contemporary school-age landscape drawings

Different from historical analyses over centuries in Study 1, Study 2 focused on rather short term ontogenetic processes. Similar to methods used by Masuda et al. (2008c) and Senzaki et al. (2014b), we had adolescent and University students in Japan and Canada create landscape drawings^3^. Furthermore, we merged these data with that of Senzaki et al.'s (2014b) work with elementary school children in order to comprehensively interpret the developmental trends of psychological tendencies in cultural products.

#### Methods

***Participants***. Students were recruited from suburban secondary schools in Japan (Iwakuni, Yamaguchi) and in Canada (St. Albert, Alberta) and Universities in Japan (Kobe University) and Canada (University of Alberta).

In the Japanese secondary school sample, there were 196 students (85 male, 107 female, 4 unspecified, *M* = 14.84, *SD* = 1.55, Range: 11–18) and was comprised of 22 seventh graders, 28 eighth graders, 42 ninth graders, 48 tenth graders, 36 eleventh graders, and 20 twelfth graders. Regarding ethnic background, all of the Japanese secondary school sample identified as Japanese and spoke Japanese as their first language. Two had lived abroad, one in China for 9 years and one in America for an unspecified number of years.

In the Canadian secondary school sample, there were 168 students (51 male, 117 female, *M* = 14.79, *SD* = 1.54, Range: 12–19). These Canadian participants were comprised of 31 seventh graders, 36 eighth graders, 24 ninth graders, 31 tenth graders, 23 eleventh graders, and 23 twelfth graders. A majority (82.74%) identified as European Canadian, 7.14% identified as biracial, 1.8% identified as East Asian, 3.57% identified as Aboriginal/Metis, 0.6% as Hispanic, and 2% as East Indian. Two students did not provide their ethnicity. Fifteen students had lived abroad, five in America, five in Europe, one in the Philippines, one in China, one in Egypt, and two in South Africa. Most of the Canadian students (99%) spoke English as their first language—two spoke other languages that were unspecified.

In the Japanese undergraduate sample, there were 75 students (38 male, 36 female, 1 unspecified, *M* = 19.71, *SD* = 1.12, Range: 18–24). All of the students identified as Japanese and spoke Japanese as their first language. Five had lived abroad for 1–2 years (two in China, one in Italy, one in Australia and one in the United States). In the Canadian undergraduate sample, there were 60 students (12 male, 48 female, *M* = 19.6, *SD* = 2.32, Range: 17–30). A majority of students (93%) identified with being European Canadian. One participant identified with being African, two as Aboriginal/Metis and one as Portuguese. All of the participants spoke English as their first language. Five had lived abroad, one in America, two in Europe, one in the Philippines and one in Brazil^4^.

***Procedure***. In classroom setting, secondary school and undergraduate students in Japan and Canada engaged in a drawing task in which they were instructed to create a landscape using a pencil on a 392 mm in width × 271 mm in height sheet of standard-sized drawing paper. Consistent with the methodology of Senzaki et al. (2014b), participants were instructed that they had to include at least one of the following: a tree, a house, a person, a horizon, and any objects they desired to draw to create their landscape artwork (see Appendix C in Supplementary Material). They were given 10 minutes to complete the task. In order to ensure that the participants understood the concept of a horizon, the experimenter defined a horizon using the following: “When you go outside, you see the sky comes down and meets the ground, and makes one line. That line is called a horizon.” Participants were also reminded that they had to complete the artwork without talking or looking at other participants' artworks. After the completion of their artwork, they were asked to fill out a simple demographic questionnaire about their gender, date of birth, ethnicity, years lived abroad (if any), and languages spoken at home.

#### Results

***Horizon height***. Consistent with previous studies (Masuda et al., 2008c; Senzaki et al., 2014b), we used the ratio of the location of the drawn horizon line to the entire frame of the drawing paper in order to determine perspective. The horizon line was assessed by two independent coders using the same guideline as in Study 1. Generally, the horizon line was determined by measuring from the bottom of the drawing paper to the drawn horizon line. The interrater agreement for the horizon height was 97% for the Japanese secondary school landscape drawings and 98% for Canadian. For the undergraduate sample, the interrater agreement was 97% for Japanese and 83% for Canadian drawings. Any discrepancies in horizon height were resolved through discussion between the coders and the primary investigator.

**Figure 2:**
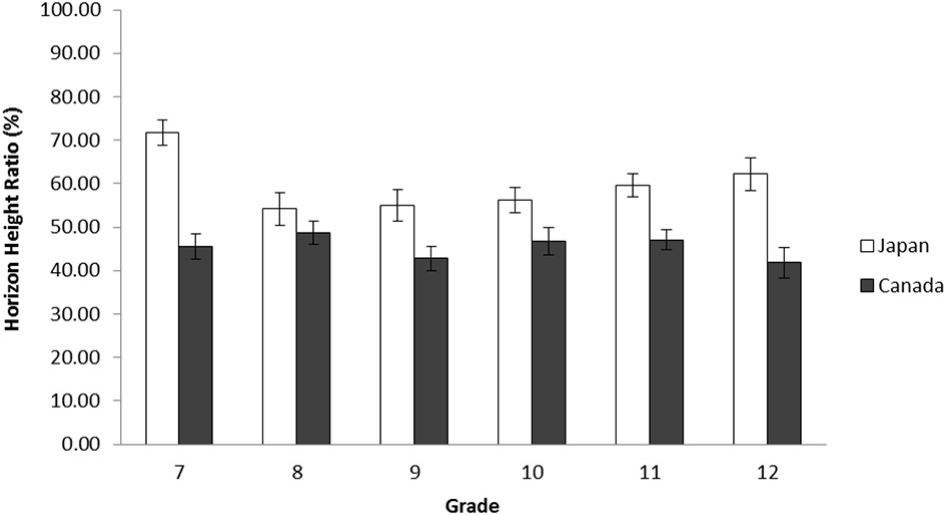
**Average horizon height ratio in percentage by grade for Japanese and Canadian adolescent landscape drawings in Study 2a**. The error bars represent standard error.

A 2(Culture: Japan vs. Canada) × 6(Grade: Grade 7, 8, 9, 10, 11, and 12) ANOVA was applied to the ratio of the horizon against the entire frame. The results indicated that there was a main effect of culture, *F*_(1, 352)_ = 56.63, *p* < 0.001, η^2^_p_ = 0.139. However, there was no main effect of grade, *F*_(5, 352)_ = 1.88, *p* > 0.009. Consistent with previous findings, Japanese secondary school students, in general, drew the location of the horizon higher (*M* = 58.62, *SD* = 19.59) than Canadians (*M* = 45.78, *SD* = 15.42), demonstrating their context-inclusiveness. There was, however, a significant interaction between culture and grade, *F*_(5, 352)_ = 2.59, *p* < 0.05, η^2^_p_ = 0.035. The simple effect analyses showed that within each grade, cultural differences were significant for Grade 7, *t*_(352)_ = 5.36, *p* < 0.001, Grade 9, *t*_(352)_ = 2.70, *p* < 0.02, Grade 10, *t*_(352)_ = 2.30, *p* < 0.02, Grade 11, *t*_(352)_ = 2.68, *p* < 0.01, and Grade 12, *t*_(352)_ = 3.79, *p* < 0.001. However, no significant cultural difference was found for Grade 8, *t*_(352)_ = 1.23, *p* > 0.20, indicating minor differences in the pattern of results (Figure 2).

**Figure 3:**
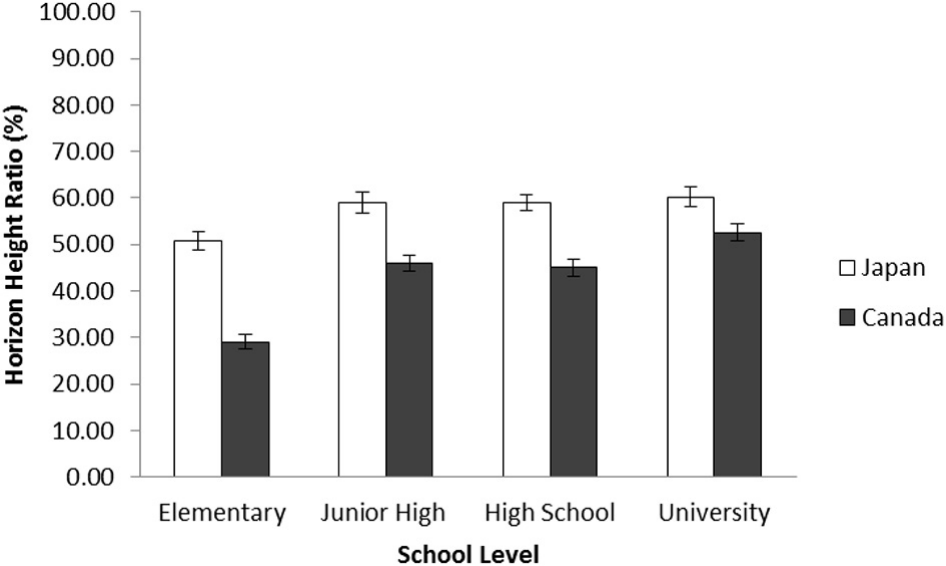
**Average horizon height ratio in percentage by school level for Japanese and Canadian landscape drawings in Study 2a**. The error bars represent standard error.

Next, to assess the generational trend of drawing, we combined and contrasted our data with elementary school data from Senzaki et al.'s (2014b) and with the university data that we collected, merging the grades according to school level. Again, a 2 (Culture: Canada vs. Japan) × 4 (School Level: Elementary, Junior High, High School, and University) ANOVA was applied to the ratio of the horizon against the entire frame. The results indicated that there was a main effect of culture, *F*_(1, 940)_ = 75.85, *p* < 0.001, η^2^_p_ = 0.075. Consistent with previous findings, Japanese, in general, drew the location of the horizon higher (*M* = 55.41, *SD* = 24.34) than Canadians (*M* = 38.21, *SD* = 21.55), demonstrating their context-inclusiveness. There was a main effect of school level, *F*_(3, 940)_ = 30.26, *p* < 0.001, η^2^_p_ = 0.088. The locations of horizon in drawings produced by junior high school students (*M* = 52.45, *SD* = 19.56), high school students (*M* = 52.94, *SD* = 18.22), and university students (*M* = 56.78, *SD* = 17.05) were significantly higher than that of elementary school children (*M* = 39.54, *SD* = 28.09), *t*s_(940)_ = 6.71, 6.94, and 8.01 all *p*s < 0.001, respectively. There was a significant interaction between culture and school level, *F*_(3, 940)_ = 4.79, *p* < 0.005, η^2^_p_ = 0.015. The simple effect analyses showed that Japanese placed the location of horizon higher in their artworks than did their Canadian counterparts in elementary schools, *t*_(940)_ = 10.50, *p* < 0.001; in junior high schools, *t*_(940)_ = 3.90, *p* < 0.001; in high schools, *t*_(940)_ = 3.97, *p* < 0.001; and in university *t*_(940)_ = 2.01, *p* < 0.05 (Figure 3).

### Study 2b: contemporary school-age landscape collages

Free drawings in Study 2a allowed us to measure one's natural expressions. However, due to the varying quality of artworks, it was difficult to count the number of objects and the area which objects occupied. To overcome these drawbacks and further scrutinize cultural differences and similarity in artworks, Study 2b used Senzaki et al.'s (2014b) collage methodology.

#### Method

***Participants***. Participants were recruited from the same suburban secondary schools in Japan (Iwakuni, Yamaguchi) and in Canada (St. Albert, Alberta). In order to see the trends in people's expression, we incorporated Senzaki et al.'s (2014b) elementary school and university data into the later analyses.

In the Japanese secondary school sample, there were 177 students (85 male, 89 female, 3 unspecified, *M* = 14.82, *SD* = 1.64, Range: 12–18). The sample was comprised of 19 seventh graders, 29 eighth graders, 39 ninth graders, 47 tenth graders, 26 eleventh graders, and 17 twelfth graders. All but one of the Japanese students identified as being Japanese and spoke Japanese as their first language. A majority had lived in Japan for their entire life.

In the Canadian secondary school sample, there were 149 students (38 male, 110 female, 1 unspecified, *M* = 14.84 years old, *SD* = 1.41, Range: 12–18). The sample was comprised of 27 seventh graders, 26 eighth graders, 26 ninth graders, 30 tenth graders, 20 eleventh graders, and 20 twelfth graders. A majority of Canadian students (81%) identified as being European Canadian, 95% spoke English as their first language and 11% had lived overseas for a short period of time^5^.

***Procedure***. In a classroom setting, secondary school students in Japan and Canada engaged in a collage task. They were instructed to create a landscape using any of thirty pre-made collage items developed by Senzaki et al. (2014b) and placing their selected pieces onto a 392 mm × 271 mm sheet of standardized laminated drawing paper using sticky tack. Similar to Study 2a, they were told to include at least one of the following: a tree, a house, a person, and a horizon, and were given the same definition of a horizon. Horizons were drawn in using a China marker (Appendix D in Supplementary Material). Participants had 15 min to create their landscape and afterward, fill out a demographic questionnaire about their gender, date of birth, ethnicity, years lived abroad (if any), and spoken languages.

#### Results

***Horizon height***. Two coders independently coded the horizon height for the collage landscape images. The interrater agreement was 99% for the Japanese secondary school collages and 93% for the Western collages. Any disagreements about horizon height were resolved by discussion between the coders and the first author.

**Figure 4:**
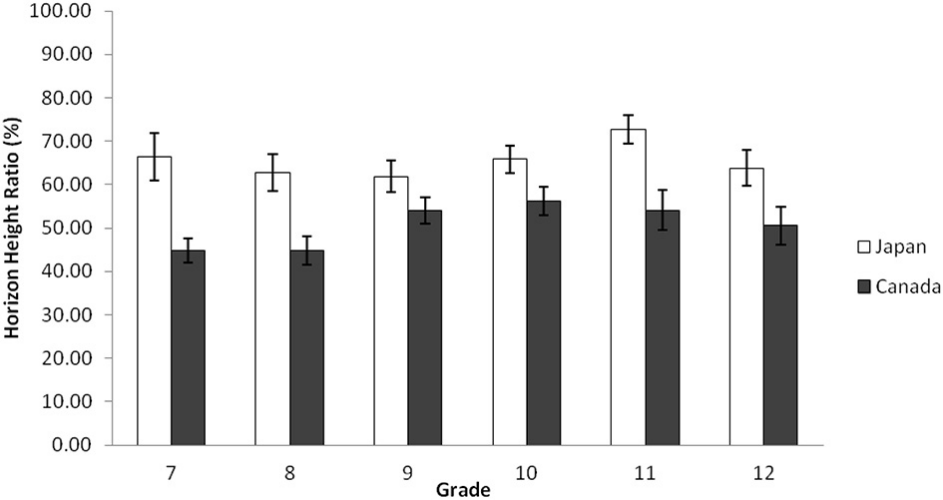
**Average horizon height ratio in percentage by grade for Japanese and Canadian adolescent landscape collages in Study 2b**. The error bars represent standard error.

Similar to Study 2a, a 2(Culture: Japan vs. Canada) × 6(Grade: Grade 7, 8, 9, 10, 11, and 12) ANOVA was applied to the ratio of the horizon against the entire frame. The results indicated that there was a main effect of culture, *F*_(1, 314)_ = 42.90, *p* < 0.001, η^2^_p_ = 0.120. However, there was no main effect of grade, *F*_(5, 314)_ = 1.66, *p* > 0.10, nor an interaction, *F*_(5, 314)_ = 1.07, *p* > 0.30. Consistent with Study 2a, Japanese secondary school students, in general, drew the location of the horizon higher (*M* = 65.34, *SD* = 21.34) than Canadians (*M* = 50.74, *SD* = 17.62), demonstrating again their context-inclusiveness. The simple effect analyses showed that within each grade, cultural differences were significant for Grade 7, *t*_(314)_ = 3.69, *p* < 0.001, Grade 8, *t*_(314)_ = 3.37, *p* < 0.001, Grade 10, *t*_(314)_ = 2.11, *p* < 0.05, Grade 11, *t*_(314)_ = 3.19, *p* < 0.005, and Grade 12, *t*_(314)_ = 2.05, *p* < 0.05. However, no significant cultural difference was found for Grade 9, *t*_(314)_ = 1.56, *p* > 0.15, indicating a minor difference in the pattern of results (Figure 4).

**Figure 5:**
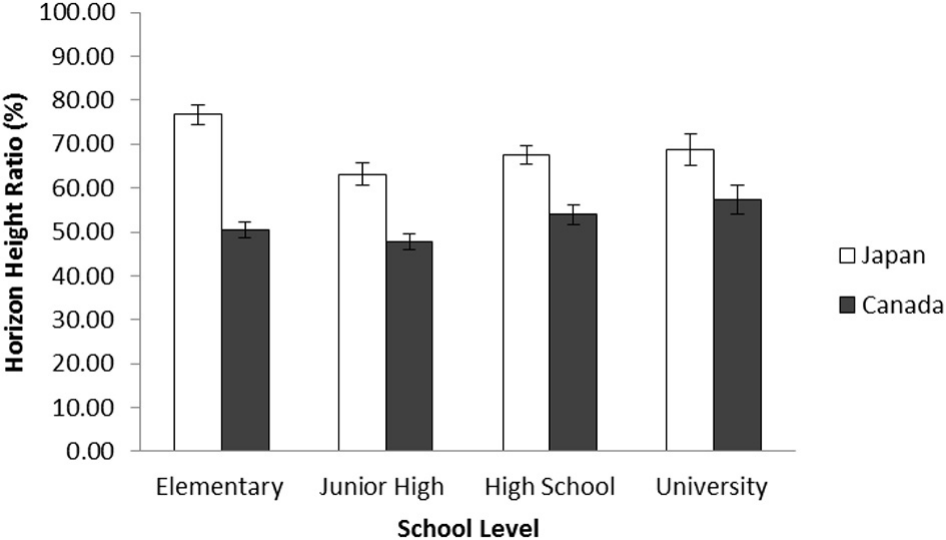
**Average horizon height ratio in percentage by school level for Japanese and Canadian landscape collages in Study 2b**. The error bars represent standard error.

After merging this data with Senzaki et al.'s (2014b) elementary school data and university data, a 2 (Culture: Canada vs. Japan) × 4 (School Level: Elementary, Junior High, High School, and University) ANOVA was applied to the ratio of the horizon against the entire frame. The results indicated that there was a main effect of culture, *F*_(1, 718)_ = 79.74, *p* < 0.001, η^2^_p_ = 0.100. Consistent with Study 2a, Japanese, in general, drew the location of the horizon higher (*M* = 70.10, *SD* = 23.39) than Canadians (*M* = 51.24, *SD* = 21.32), demonstrating their context-inclusiveness. There was also a main effect of school level, *F*_(3, 718)_ = 5.06, *p* < 0.002, η^2^_p_ = 0.021. These results are, however, qualified by a significant interaction between culture and school level, *F*_(3, 718)_ = 4.82, *p* < 0.002, η^2^_p_ = 0.020. The simple effect analyses showed that Japanese placed the location of horizon higher in their artworks than did their Canadian counterparts in elementary school, *t*_(718)_ = 10.58, *p* < 0.001; in junior high school, *t*_(718)_ = 4.47, *p* < 0.001; in high school, *t*_(718)_ = 3.83, *p* < 0.001; and in university, *t*_(718)_ = 2.28, *p* < 0.05, showing a robust cultural variation in the horizon height. In addition, Japanese elementary school students placed the horizon significantly higher than did their junior high school, high school, and university counterparts, *t*s_(718)_ = 4.47, 3.10, 2.01, *ps* < 0.001, 0.002, 0.05, respectively, whereas Canadian junior high school students placed the horizon significantly lower than did university students, *t*_(718)_ = 2.14, *p* < 0.05, indicating minor differences in patterns (Figure 5).

**Figure 6:**
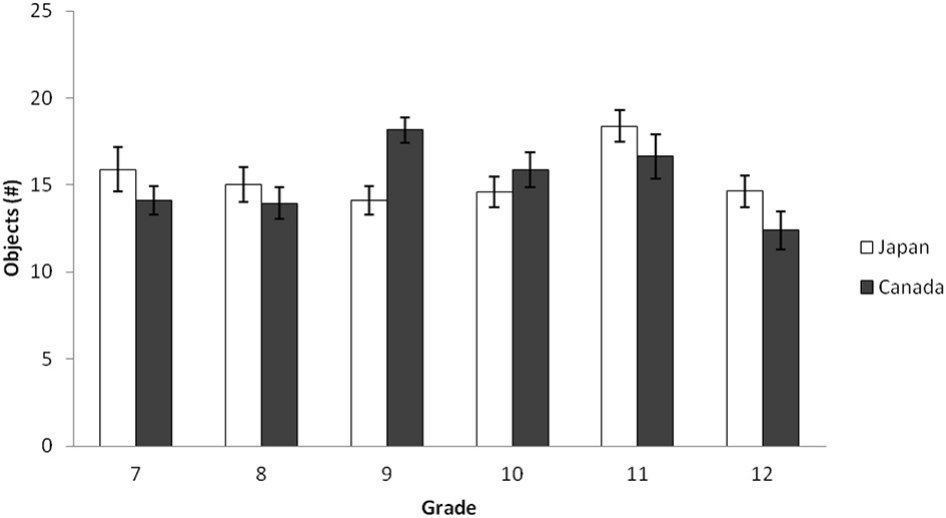
**Average number of objects by grade for Japanese and Canadian adolescent landscape collages in Study 2b**. The error bars represent standard error.

***Number of objects***. Two coders independently counted the number of objects in each collage landscape. The interrater agreement was 99% for the Japanese secondary school collages and 95% for Canadian. A 2(Culture: Canada vs. Japan) × 6(Grade: Grade 7, 8, 9, 10, 11, and 12) ANOVA was used to determine context-sensitivity for secondary school students through the number of objects in the landscape scene. There was a main effect of grade, *F*_(5, 314)_ = 3.23, *p* < 0.05, η^2^_p_ = 0.049, and an interaction between culture and grade, *F*_(5, 314)_ = 3.16, *p* < 0.01, η^2^_p_ = 0.048. Unlike the horizon height data, there was no main effect of culture for the number of objects in collage landscapes, *F* < 1, *ns*. The simple effect analyses revealed that the pattern in the number of objects was reversed for Grade 9 where Canadians had more objects than Japanese, *t*_(314)_ = 3.12, *p* = 0.002 (Figure 6).

**Figure 7:**
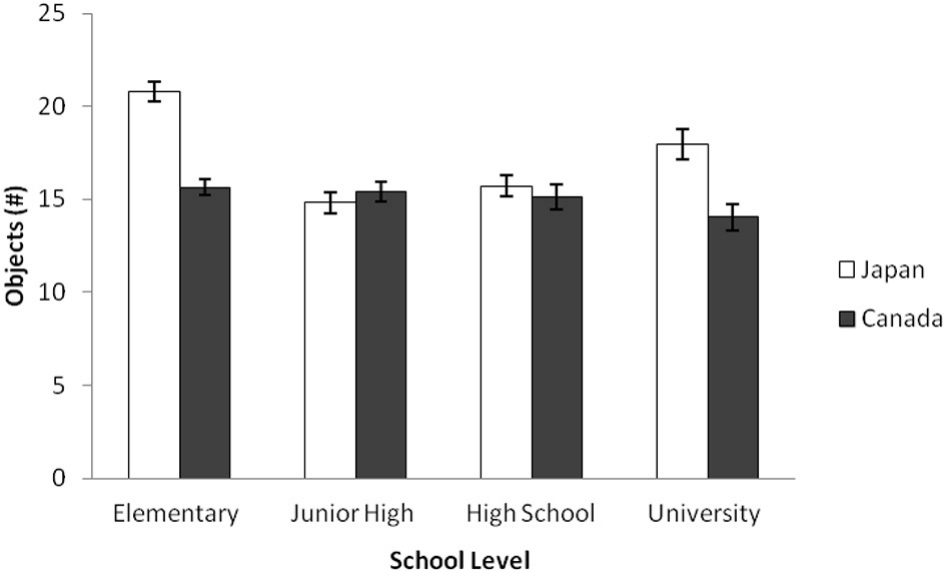
**Average number of objects by school level for Japanese and Canadian landscape collages in Study 2b**. The error bars represent standard error.

Following merging this data with Senzaki et al.'s (2014b) elementary school data and university data, a 2 (Culture: Canada vs. Japan) × 4 (School Level: Elementary, Junior High, High School, and University) ANOVA was applied to the ratio of the horizon against the entire frame. The results indicated that there was a main effect of culture, *F*_(1, 718)_ = 22.47, *p* < 0.001, η^2^_p_ = 0.030, and of school level, *F*_(1, 718)_ = 15.17, *p* < 0.001, η^2^_p_ = 0.060. These patterns were qualified by an interaction of culture and school level, *F*_(1, 718)_ = 11.59, *p* < 0.001, η^2^_p_ = 0.046. The simple effect analyses revealed that elementary school and university data showed culturally dominant patterns—Japanese placed more objects in their artworks than did Canadians, *t*s_(718)_ = 8.07 and 3.01, *p*s < 0.001 and 0.005, respectively. The junior high school and high school data, however, did not show any cultural differences regarding the number of objects, *F*s < 1, *ns*. In Japanese data, the number of objects in junior high school data was significantly smaller than these of elementary school and university data, *t*_(718)_ = 7.70, *p* < 0.001, and *t*_(718)_ = 2.89, *p* < 0.005, respectively. The same patterns were observed for high school data, *t*_(718)_ = 6.62, *p* < 0.001, and *t*_(718)_ = 2.08, *p* < 0.005, respectively. In contrast, the patterns were rather constant in Canadian data (all *p*s are *ns*). In sum, Japanese adolescents' patterns regarding the number of objects were different from the dominant patterns observed in elementary school and university data, and similar to that of Canadian data, suggesting a substantial drift during this ontogenetic period only for Japanese (Figure 7).

**Figure 8:**
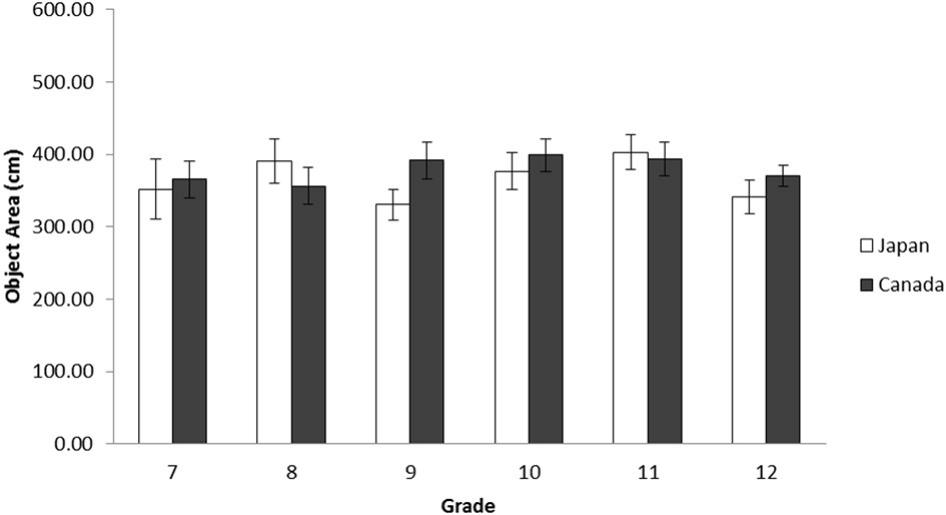
**Average area covered by objects in centimeters by grade for Japanese and Canadian adolescent landscape collages in Study 2b**. The error bars represent standard error.

***Object area***. As another measure of context-sensitivity, we also determined the amount of space used in the created landscapes through the area occupied by the collage pieces on the frame. A 2(Culture: Japan vs. Canada) × 6(Grade: Grade 7, 8, 9, 10, 11, and 12) ANOVA was applied to the object area. The results, however, indicated that there was no main effect of culture or grade, nor an interaction, *F*s < 1, *ns*. Therefore, there was no difference in the area covered by objects for Japanese and Canadian secondary school students (Figure 8).

**Figure 9:**
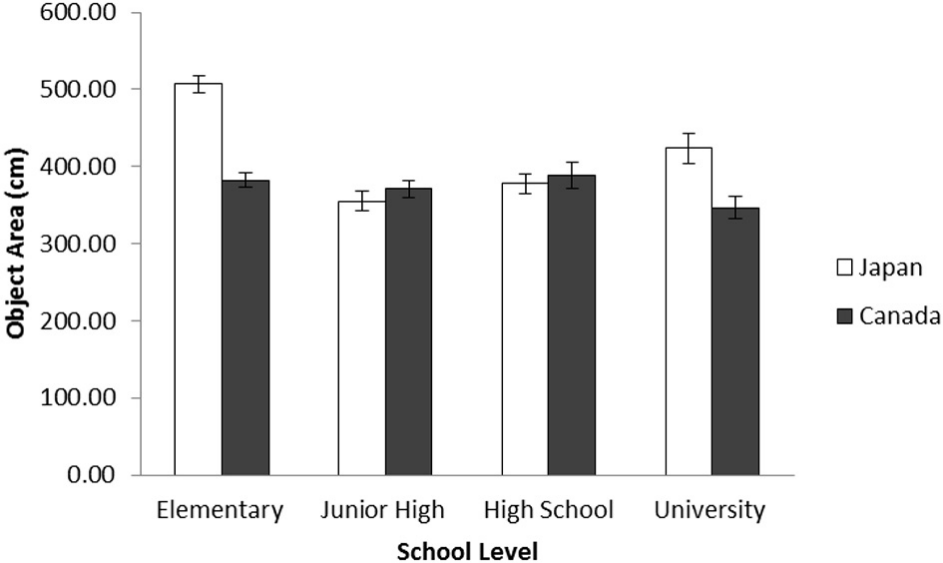
**Average area covered by objects in centimeters by school level for Japanese and Canadian landscape collages in Study 2b**. The error bars represent standard error.

After merging our data with Senzaki et al.'s (2014b) elementary school data and university data, a 2 (Culture: Canada vs. Japan) × 4 (School Level: elementary, junior high, high school, and university) ANOVA was applied to the object area. The results indicated that there was a main effect of culture, *F*_(1, 718)_ = 17.24, *p* < 0.001, η^2^_p_ = 0.023, and of school level, *F*_(1, 718)_ = 19.01, *p* < 0.001, η^2^_p_ = 0.074. These patterns were qualified by an interaction between culture and school level, *F*_(1, 718)_ = 16.61, *p* < 0.001, η^2^_p_ = 0.065. The simple effect analyses revealed that elementary school and university data showed culturally dominant patterns, Japanese having used more area in their artworks than did Canadians, *t*s_(718)_ = 8.89 and 2.72, *p*s < 0.001 and 0.01, respectively. The junior high school and high school data did not show any cultural differences regarding the number of objects, *F*s < 1, ns. This pattern was observed only in Japanese data, as the area of objects in junior high school data was significantly smaller than that of elementary school and university data, *t*_(718)_ = 8.88, *p* < 0.001, and *t*_(718)_ = 2.89, *p* < 0.005, and that of high school data was significantly smaller than that of elementary school data, *t*_(718)_ = 7.64, *p* < 0.001, and marginally smaller than that of university data, *t*_(718)_ = 1.94, 0.05 < *p* < 0.10. In contrast, the patterns were rather constant in Canadian data (all *p*s were *ns*). Similar to the data of the number of objects, Japanese adolescents' patterns regarding the area of objects were different from the dominant patterns observed in elementary school and university data, and similar to these of Canadian data, again suggesting a substantial drift during this ontogenetic period only for Japanese (Figure 9).

#### Discussion

Study 2a and 2b's results with Senzaki et al.'s (2014b) data suggests that, although there are some fluctuations (e.g., Grade 8 data in Study 2a and Grade 9 data in Study 2b), cultural variations in the location of horizon drawn by Japanese and European Canadian are robust: in general, Japanese consistently place the horizon higher than their European Canadian counterparts. Therefore, the “resilience to change” hypothesis was supported for the location of horizon. However, the results of the number of objects and the area occupied by the objects suggests that, although Japanese elementary school children and undergraduate students were more likely than their European Canadian counterparts to include more pieces of information, and use more space to place pieces of information, the patterns of junior high school and high school students were almost equal across cultures (in Grade 9, Japanese demonstrated less context-sensitivity than Canadians), and the changes in expressions were substantial. By looking at the trends, we conclude that during adolescence, Japanese's scores for these two variables decreased whereas Canadian scores in general remained constant. Therefore, the “cultural drift” hypothesis was supported for the number of objects and the area occupied by the objects only for Japanese adolescents, showing a curvilinear trend—cultural variations in drawing emerged during elementary school data, disappeared during secondary school data, and reappeared in undergraduate data.

Why did Japanese adolescents' artworks become similar to that of their European Canadian counterparts? We assume the influence of Western popular cultures would be strong among Japanese adolescences. Young children of a given culture set their life task to internalize dominant cultural meaning systems into their behavior thorough interaction with their caregivers, teachers, and those who sustain the dominant norms. However, adolescent members are generally active in their seeking of alternative values, and developing sub-cultures within society, while searching for new, unfamiliar, and cool expressions. Therefore, they are very much susceptible to popular cultures developed in Western societies. Kinsella (1995) suggests that following the introduction of Disney in the early 20th century, Japanese adolescents were receptive to “cute” European styles because it contrasted with dated products in traditional Japanese society. Teenagers' rebellious attitude against their parents and teachers' generation could be another facilitator of cultural drift (Kroger, 2007). However, during post-secondary education, undergraduate students may resume the dominant norms to become mature adults. If so, it is not surprising that their artistic expressions again show culturally dominant patterns by placing the horizon high, using more objects, and occupying more space in the visual field.

Although only a single observation suggested it, Canadian Grade 9 students in Study 2b placed a significantly larger number of objects in their artworks than did Japanese. We speculate that, although the effect is minor, recent trends of East Asian popular cultures may start to be consumed by Canadian adolescents. For example, *manga* (Japanese comics) has become internationally popular and is readily available in bookstores across North America, the first issue of *Shonen Jump* selling out at 250,000 copies (Wong, 2006). In fact, such East Asian products are recently easily accessible through the internet when compared to a decade ago. Although we do not know how much manga may influence landscape art, the depiction of art similar to that of Japanese suggests that North American adolescents may be more willing to access, be influenced by, and be receptive to emulating the work of other cultures.

In sum, ontogenetic data of people's artistic expressions in Study 2a and b demonstrated that, although dominant patterns of artistic expressions in general exist, the results of Japanese adolescent data show evidence of cultural drift.

## General discussion

In an extension of Masuda et al.'s (2008c) and Senzaki et al.'s (2014b) findings, we examined Japanese and Western historical landscape masterpieces from the 17 to 20th century and Japanese and Canadian adolescents' landscape artworks in order to comprehensively examine the trends regarding perceptual tendencies in both history and development. This research is among the first to use both historical and ontogenetic data in order to thoroughly examine both persistence of culturally-dominant expressions and substantial cultural drifts. The results of historical data in Study 1 suggest that although cultural variations in masterpieces arts were stable for 250 years, a major cultural change like the Meiji Restoration bidirectionally changed both Japanese and Westerners' expressions. Nonetheless, culturally dominant expressions emerged again due to persistence of cultural meaning systems in the social structure while the results of ontogenetic data in Study 2a and b suggest that on top of robust cultural variation in artworks, adolescent Japanese expressions are somewhat similar to their European Canadian counterparts; however, culturally dominant expressions emerged again after adolescence.

## Implications

There are several implications for this research. First, investigating cultural products is an important and useful method to understand how culture is both created and maintained. Visual representations in particular are a rich medium and a snapshot in time in order to examine how psychological tendencies create and maintain culture. Although we focused only on landscape arts, there are many other media which can be a target of analyses such as movies, TV programs, flyers, and magazine ads (Masuda et al., 2012b).

Second, by utilizing the historical framework, the current paper demonstrated cultural drifts exist across time on top of the robust cultural variations in artistic expression. Future studies in psychology should thus integrate and explore more data in order to elucidate dynamic patterns regarding whether the human psyche is indeed changing according to an historical event and how it influences human psyche in a given culture, and what to possibly anticipate for the future. These investigations would not be actualized by experimentation commonly used in psychology as the current findings suggest the necessity of further collaborations with the fields of humanities such as philosophy, history, and art.

Third, the current studies suggest that cultures are not isolated from others—rather, they mutually influence each other through borrowing, imitating, and modifying foreign products to incorporate them into their culture, which is commonly observed in history. Our historical data suggest that is the case. Furthermore, researchers have recently been acknowledging that, with globalization processes, our psyche are inevitably influenced by other cultures (Chiu and Hong, 2006; Chiu et al., 2011), particularly in adolescence (Jensen, 2003). Our ontogenetic data demonstrated that Japanese adolescents behaved differently than their elementary school and adult counterparts, showing a more Western pattern of aesthetics regarding the number of objects, and the area of objects. We assume that this developmental stage may have potential to drift away from culturally normative behavior. However, to date, little cross-cultural research focuses on adolescents' mentality. Future research should scrutinize the mechanism of ontogenetic transition during adolescence in relation to cultural tendencies.

## Limitations and future directions

Our findings provide the first evidence of both historical and ontogenetic data which show cultural stability and drifts in people's artistic expressions. Nonetheless, to more comprehensively examine cultural variations in development as a function of historical circumstances, further examination is mandatory. First of all, a longitudinal study both within and between generations should be conducted in future research. For example, a future study may want to examine cultural products from different cohorts of East Asian and North American elementary school participants. By having a new cohort and following up with the previous one over set periods of time, cultural drift within individuals can be closely examined.

In addition, the current studies did not assess whether or not Japanese students had taken Western cultural courses or if Canadians had experience in East Asian cultural studies, and if these students had exposure to different perspectives for landscape art in their optional art classes. In future studies it may be useful to quantify whether or not participants are accessing or have exposure to artwork from other cultures. Careful examination of differences in educational systems may also further refine the quality of data.

Finally, and most importantly, the data were collected from only one or two schools in a specific area of each respective culture. Although we believe that students at the selected schools represented average adolescent and young adult behaviors in each culture, and although cultural psychologists conventionally collect data from a single research field per each culture, the generalizability of findings should be tested in future replication research. In fact, some studies have reported within-cultural differences in cognition and perception (e.g., Snibbe and Markus, 2005; Kitayama et al., 2006). Ideally, it is advisable to conduct a more comprehensive, population-level study in collaboration with other researchers, which will reinforce the validity of the findings.

## Conclusion

Culture is a dynamic process. Beyond the static perspective, the current findings provide the evidence of the effectiveness of historical and ontogenetic analyses of a cultural phenomenon (artistic expressions) and addressed the issue of cultural change under the rubric of cultural drift. Future research should further apply these approaches, alongside phylogeny, to elucidate dynamic relationships between culture and psychology.

### Conflict of interest statement

The authors declare that the research was conducted in the absence of any commercial or financial relationships that could be construed as a potential conflict of interest.

## Footnotes

^1^Japanese landscape paintings from the 17th century usually lacked a specific date the painting was made or a distinguishable horizon.

^2^As for the statistical notations, we followed the logic of Howell's book “Statistical Methods for Psychology” (2007), and Mori and Yoshida (1990). Based on their logic, we ran simple effect analyses and reported the results using the mean square error and degree of freedom from the omnibus test.

^3^In Masuda et al. (2008c), landscape drawing data were collected from University students; however, the East Asian participants consisted of international students. Furthermore, the data were collected in 2001 and conditions in the method differed, such as having to draw a river [which was excluded in Senzaki et al.'s (2014b) study] and the usage of a smaller paper size. Therefore, to maintain consistency and to be able to determine developmental patterns through this cross-sectional design, we re-collected drawing data from University students.

^4^Students from both cultural groups who did not follow instructions, such as missing a required item (*n* = 19) or not taking the task seriously (e.g., Drawing inappropriate material, *n* = 14) were excluded from these numbers and the subsequent analysis. Given that children generally learn about the concept of a horizon from Grade 2 (Senzaki et al., 2014b), any drawings that did not demonstrate an understanding of a horizon (such as having floating objects or two horizon lines) were also not included in the analysis (*n* = 9).

^5^Similar to Study 2a, students in Study 2b in both cultural groups who did not follow instructions (*n* = 28), take the task seriously (*n* = 6) or understand the concept of a horizon (*n* = 27) were excluded from the analysis.
