# Supplementary material for: Examining cultural drifts in artworks through history and development: cultural comparisons between Japanese and western landscape paintings and drawings
Source: Front Psychol. 2014 Sep 19;5:1041. doi: 10.3389/fpsyg.2014.01041 (PMC4168670; doi:10.3389/fpsyg.2014.01041)
Supplement: Supplementary file 1 [file DataSheet1.DOCX]

# Appendix A: Accessed Museums and Books

#
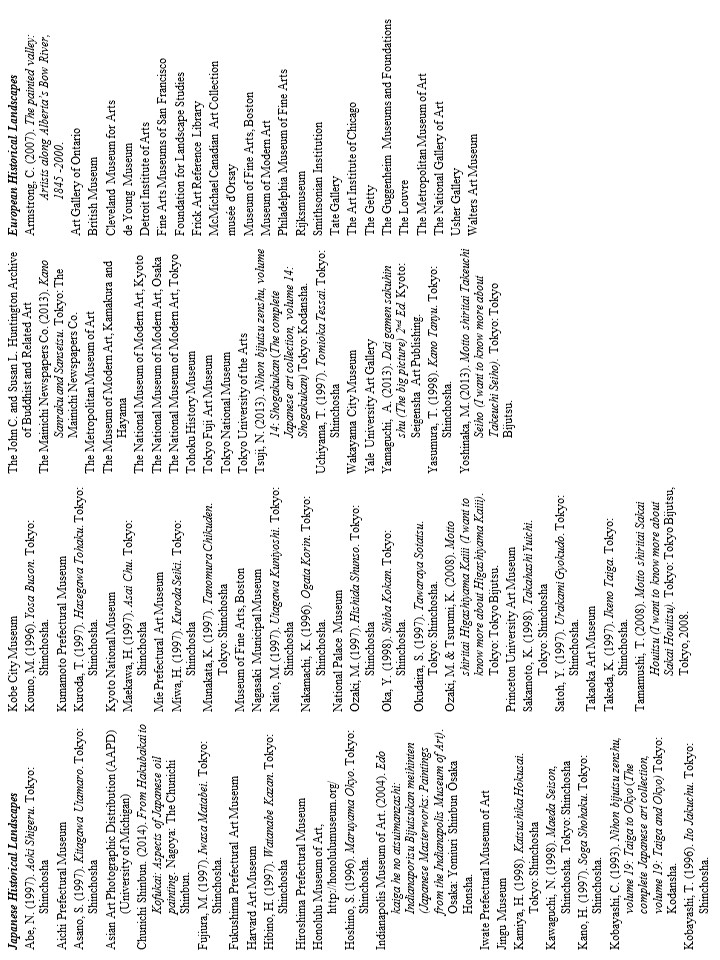


# Appendix B: Horizon Measuring Guide

1. If it is a standard horizon (a horizontal line dividing sky and land), measure from the bottom of the page to the highest point on the line and the lowest.


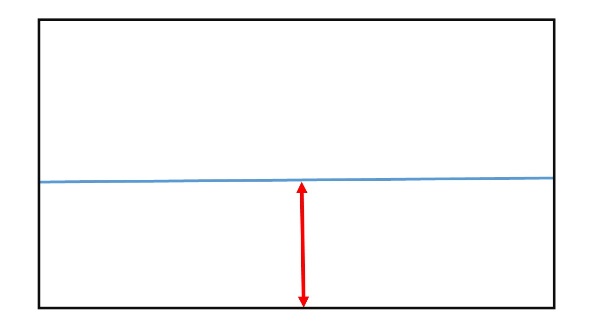


1. If there are mountains, measure:
   1. The highest dip or descent and the lowest.


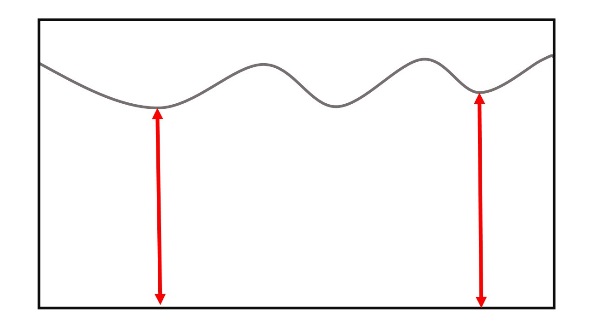


- 1. If there is a horizontal line below the mountain, measure the lowest point on the horizontal line and the highest dip or descent


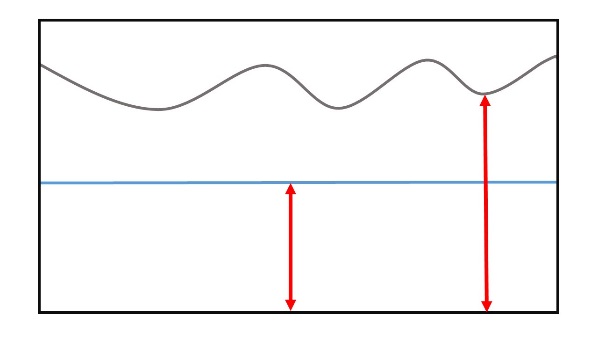


1. If there are cliffs are in the foreground, measure only what is in the background.


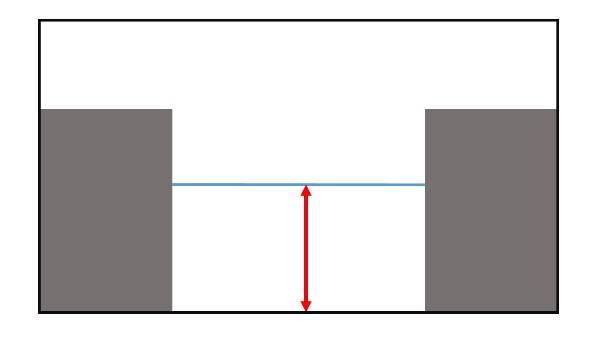


**Appendix C: Samples of Landscape Drawings**


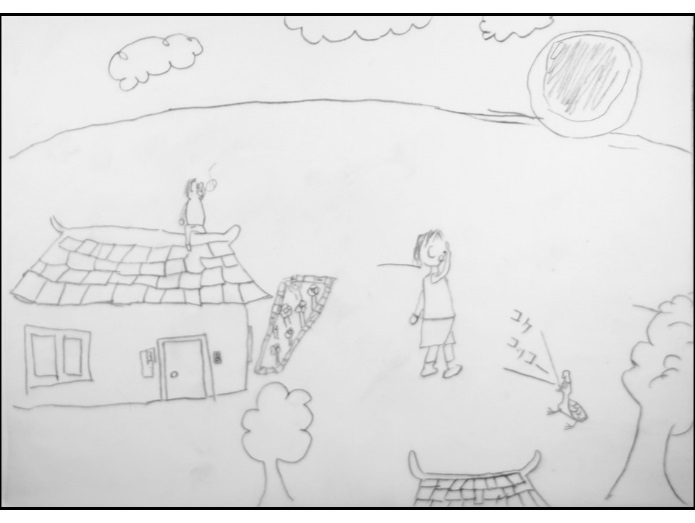

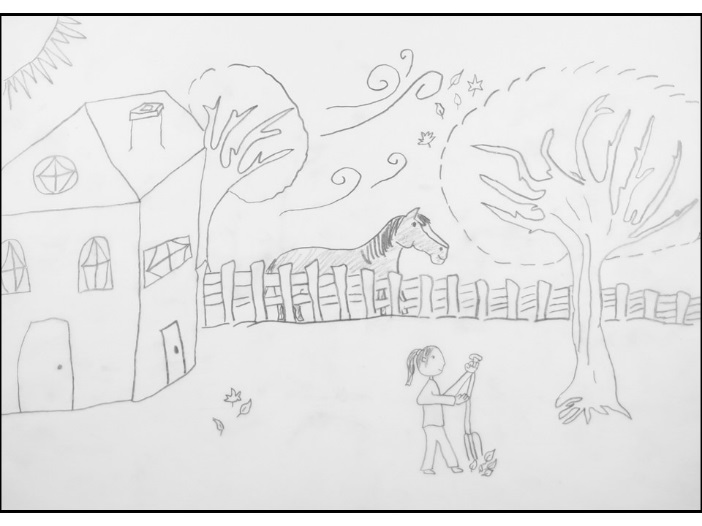


Grade 7 (Japanese) Grade 7 (Canadian)


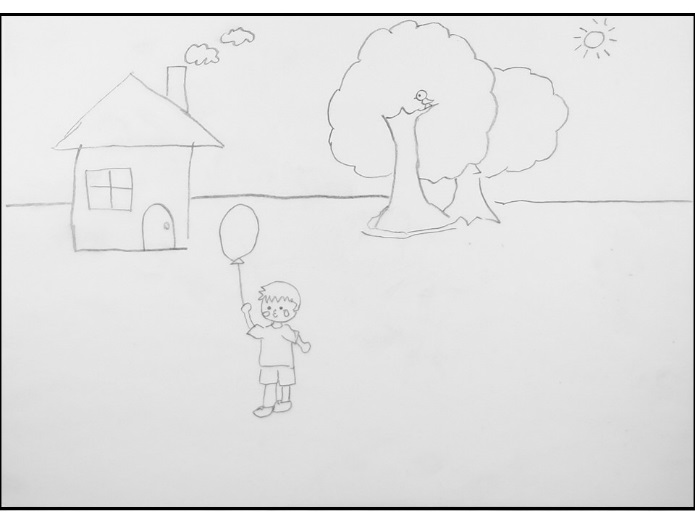

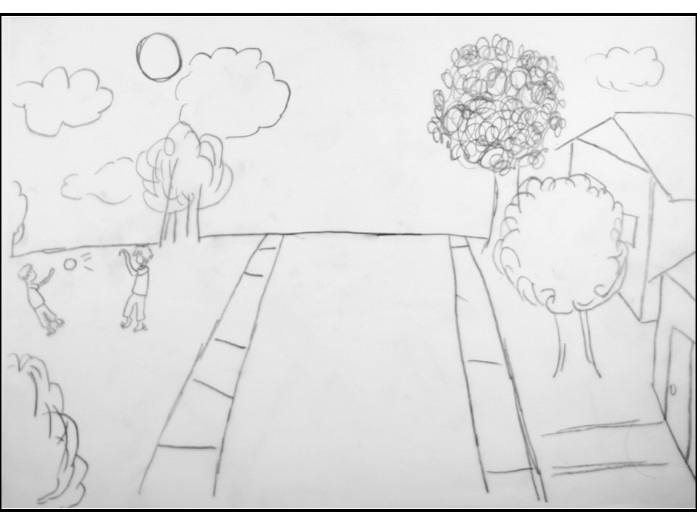


Grade 10 (Japanese) Grade 10 (Canadian)


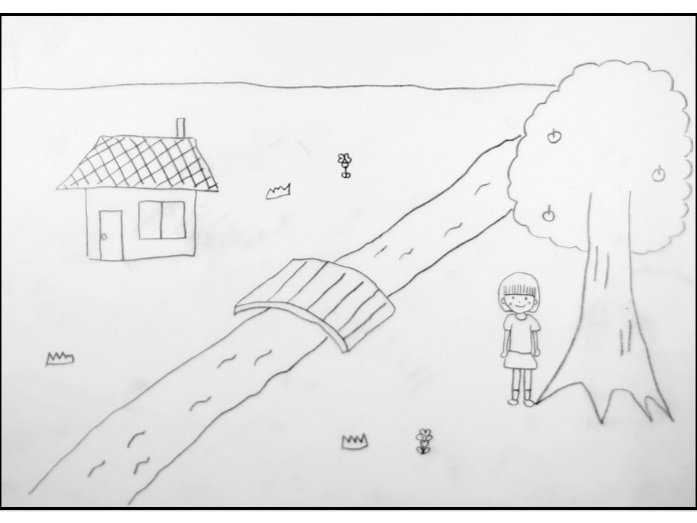

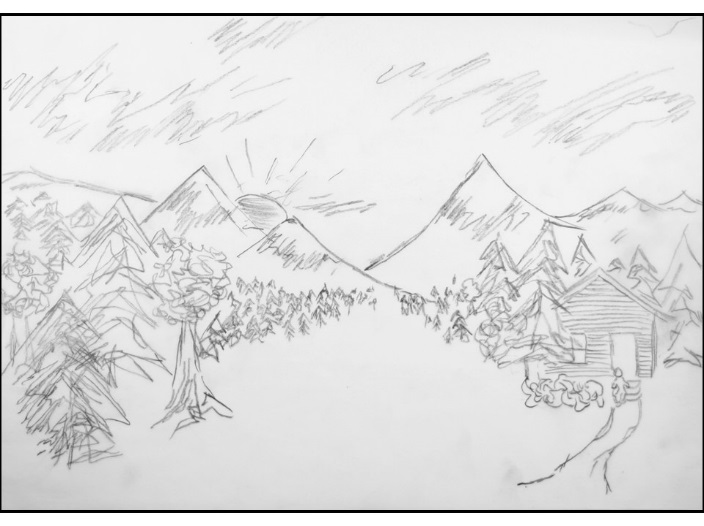


University (Japanese) University (Canadian)

# Appendix D: Samples of Landscape Collages


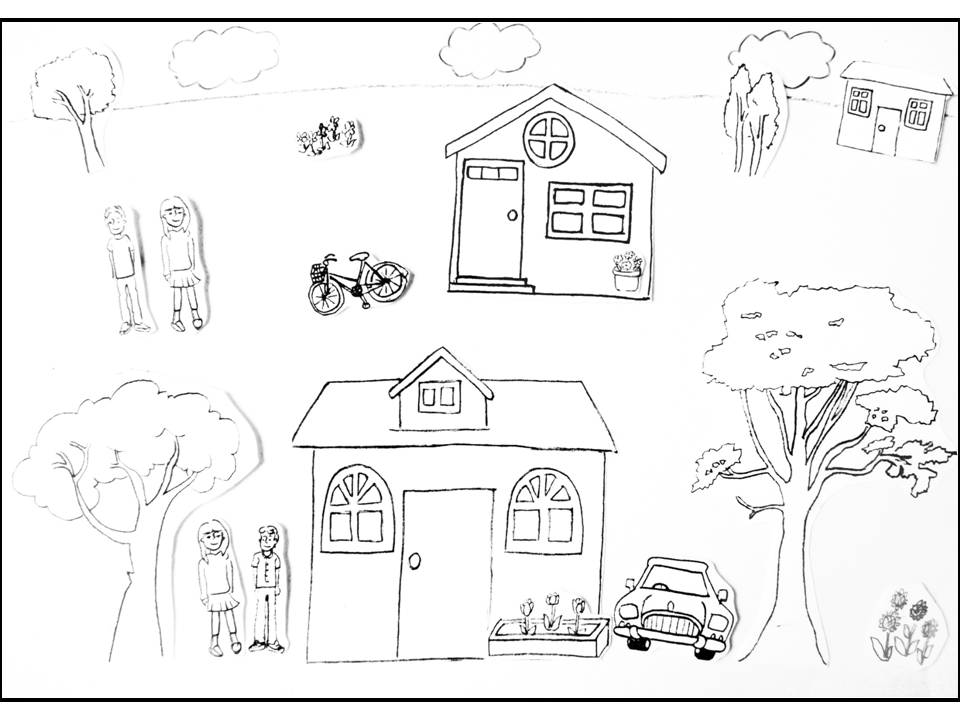

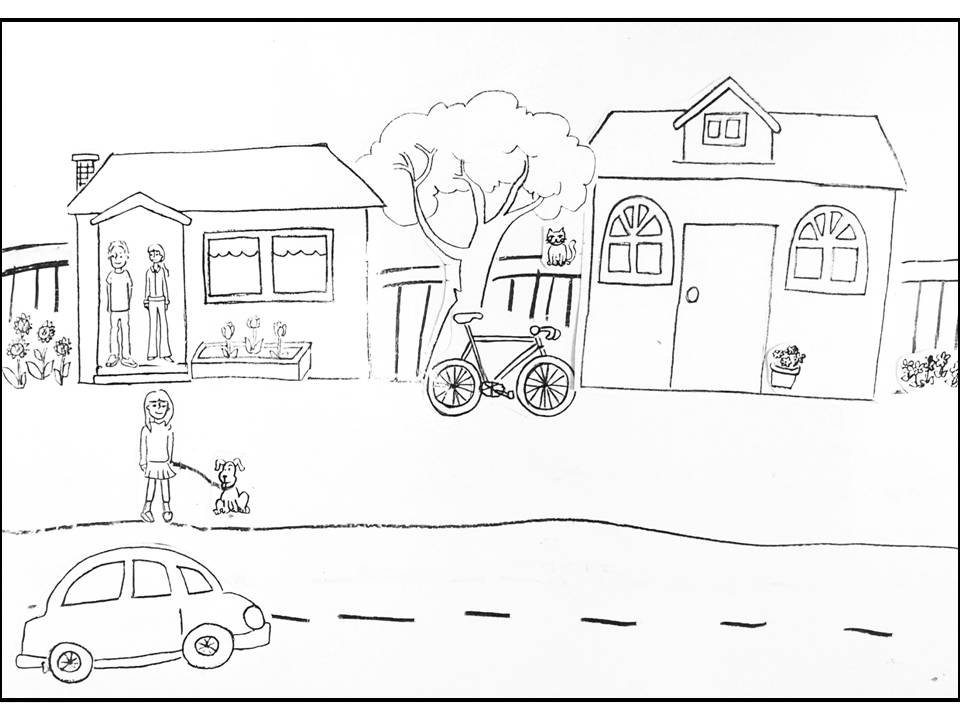


Grade 7 (Japanese) Grade 7 (Canadian)


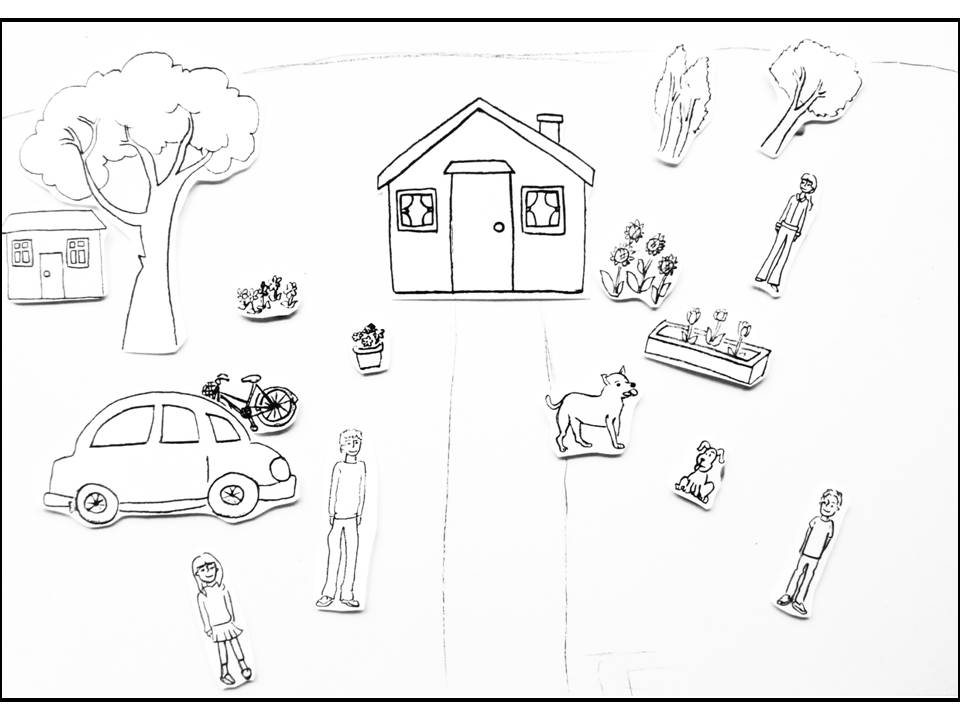

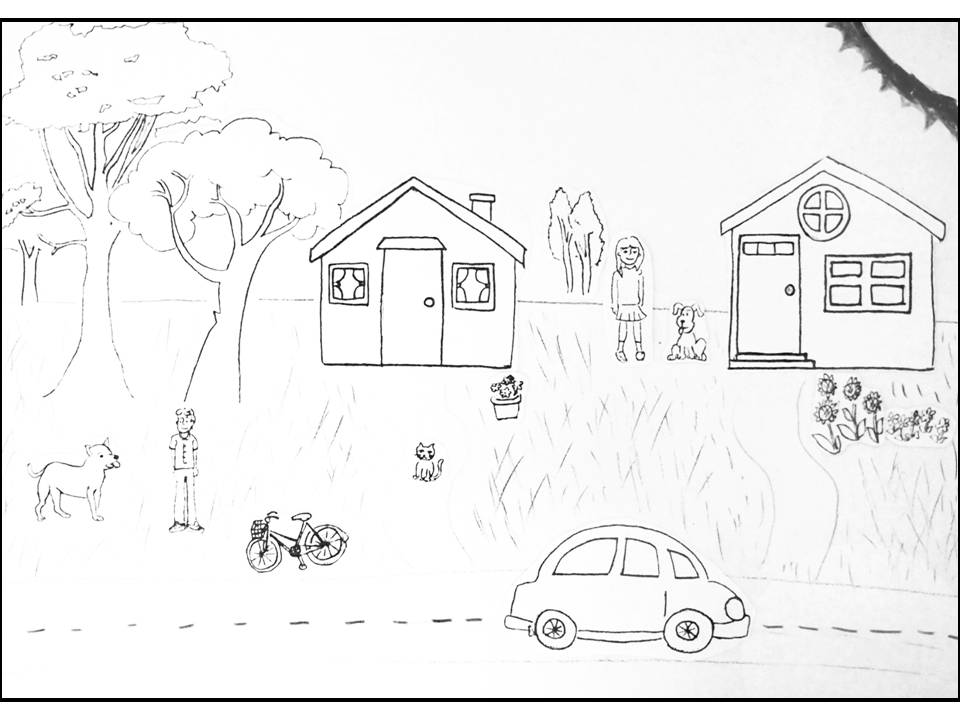


Grade 10 (Japanese) Grade 10 (Canadian)
